# Insights on brain functions in burning mouth syndrome

**DOI:** 10.3389/fnsys.2022.975126

**Published:** 2022-09-02

**Authors:** Cosmin Dugan, Ioanina Parlatescu, Maria Dobre, Raluca Ema Pîrvu, Elena Milanesi

**Affiliations:** ^1^Carol Davila University of Medicine and Pharmacy, Bucharest, Romania; ^2^Department of Oral Pathology, Faculty of Dental Medicine, Carol Davila University of Medicine and Pharmacy, Bucharest, Romania; ^3^Victor Babes National Institute of Pathology, Bucharest, Romania

**Keywords:** burning mouth syndrome, glossodynia, neuroimaging, fMRI, hippocampus

## Introduction

Burning mouth syndrome (BMS) is a multifactorial disease characterized by chronic persistent oral mucosa pain with normal clinical appearance, biological investigations, and sensory testing (International Classification of Orofacial Pain, 1st edition (ICOP), 2020). The International Headache Society defined it as “an intraoral burning or dysaesthetic sensation, recurring daily for more than 2 hours/day over more than three months” (International Headache Society, 2018). Additional clinical terms used are glossodynia, glossopyrosis, oral dysesthesia, stomatopyrosis, stomatodynia, primary burning mouth syndrome and lately burning mouth disease (Van, 2021). The disorder previously named secondary BMS has a local or systemic cause, so it is excluded from BMS (International Classification of Orofacial Pain, 1st edition (ICOP), 2020).

The prevalence of BMS varies in epidemiological studies between 1.73% in the general population and almost 8% in clinical patients, most likely affecting postmenopausal women over 50 years (Wu et al., 2021). The reported gender differences ratio of 3:1 more women to males can be accounted by biological, psychological, and behavioral causes (Wu et al., 2021).

BMS symptoms usually appear in the morning, grow during the day and, reach their peak evening (Grushka et al., 2002). In the last decades, BMS has been associated with persistent anxiety or depression (Forssell et al., 2002; Scala et al., 2003) and hormonal changes mainly menopause-related (Khan et al., 2014).

As the diagnostic and therapeutic approach of BMS raises difficulties, the neuropathic peripheral and central pathophysiological mechanisms gain more ground compared to psychological disorders (Orliaguet and Misery, 2021). The absence of direct damage signs to the somatosensory nervous system in BMS promotes the idea of its dysfunction and in the brain network (Carreño-Hernández et al., 2021). Furthermore, BMS shares several characteristics with other chronic primary pain conditions in terms of nociplastic pain (pain in the symptomatic areas, and may be accompanied by sensitized pain perception—allodynia or hyperalgesia) (Imamura et al., 2020).

The higher prevalence of BMS in postmenopausal women and the significant increased expression of transient receptor potential vanilloid 1 (TRPV1) detected in BMS epithelium are arguments for estrogen implications (Seol and Chung, 2022). TRPV1 is a nociceptive receptor modulated by estrogen and considered a chronic pain mediator. Estrogen has a two-hit effect on TRPV1: increased estrogen upregulates TRPV1 and deficient estrogen downregulates nerve growth factor which increases TRPV1 (Nagamine, 2022). These interactions explain the gender association, pain perception and could be targeted by therapies.

The BMS related neural peripheral conditions are abnormalities of the small A taste afferents or peripheral trigeminal nerves or lower nerve density or chorda tympani hypofunction (Eliav et al., 2007; Orliaguet and Misery, 2021). These features recommended the use of functional Magnetic Resonance Imaging (fMRI) for diagnosis and stratification, given the limited capacity that has been demonstrated in most somatic and psychological investigations.

Recent advances in neuroimaging by non-invasive measurements accurately decode a human's conscious experience based on brain activity. As in the visual field perception, this “brain reading” helps to reveal how individual experiences are coded, the same approach to other types of mental states can be done (Logothetis, 2008).

To date, there are few studies analyzing fMRI in both BMS patients and healthy individuals with slightly different results (Albuquerque et al., 2006; Tan et al., 2019; Kurokawa et al., 2021).

In this brief opinion article, we focus on the use of fMRI application in burning mouth syndrome as a major milestone for an enigmatic, multifactorial disease.

## Functional brain imaging by magnetic resonance imaging

fMRI visualizes cortical activity by spatially specific hemodynamics (alterations of the blood flow) and neurovascular connectivity. Based on the imaging of calcium concentrations, it detects signals that reflect neuronal activity (Jasanoff, 2007). The mechanisms used are the blood oxygenation level-dependent effect, measurement of cerebral blood flow changes with arterial spin labeling, intravoxel incoherent motion, and cerebral blood volume alterations (Harel et al., 2006).

The fMRI scan enables the evaluation of sensory processing or action controls, as well as the neural mechanisms of recognition and memory. The fMRI studies based on the hemodynamic response explore both functional and cognitive topographic segregation associated with tasks or cognitive stimuli (Logothetis, 2008).

## Central neurologic aspects and fMRI in burning mouth syndrome

The pain-responsive cerebral matrix comprises the somatosensory areas which include insula, thalamus, anterior cingulate cortex, prefrontal area, and the associated areas. These are the primary and supplementary motor areas, posterior parietal area, posterior cingulate cortex, basal ganglia, hypothalamus, amygdala, cerebellar peduncle nuclei, and periaqueductal gray matter (Kurokawa et al., 2021). Measuring brain function in BMS patients and controls brings some insight into their brain activity. The main results of nine fMRI studies for BMS patients are presented in Supplementary Table 1. All these studies include a relatively small number of patients leading to limited conclusions.

Decoding the fMRI changes in 26 BMS patients compared to 27 controls, Tan et al. reported decreased gray matter volume (GMV) in the bilateral ventromedial prefrontal cortex (VMPFC) accompanied by an enhanced functional connectivity between this area and the bilateral amygdala. BMS patients had higher depression and anxiety levels and the lower GMV was inversely correlated with the severity of BMS. The same authors also indicated that the levels of functional connectivity between the bilateral VMPFC and amygdala seem to be in connection with the BMS duration (Tan et al., 2019).

Albuquerque and collaborators revealed variations in location and extent of brain activation between BMS female patients compared to a control group during thermal painful stimulation. Patients with BMS showed less volumetric activation throughout the entire brain and greater fractional signal changes of the right anterior cingulate cortex and bilateral precuneus. The cerebellum, left lingual gyrus, right precentral gyrus, bilateral thalamus, and right middle frontal gyrus displayed larger fractional signal changes in the control group (Albuquerque et al., 2006).

Khan et al. observed in 9 BMS patients a higher GMV and lower fractional anisotropy in the right hippocampus and a lower GMV in the medial prefrontal cortex (mPFC). In different states of pain/burning, increased functional connectivity patterns were found between mPFC and anterior cingulate cortex, occipital cortex, VMPFC and bilateral hippocampus/amygdala in the afternoon compared with the morning session (Khan et al., 2014).

Another study examined the structural changes in idiopathic BMS patients compared to dysgeusic patients and healthy subjects (Sinding et al., 2016). Anterior and posterior cingulate gyrus, lobules of the cerebellum, insula/frontal operculum, inferior temporal area, primary motor cortex, and dorsolateral prefrontal cortex were identified as the six affected regions of the eight investigated. Pain intensity was found to correlate with GMV in the anterior cingulate gyrus, the cerebellum lobules, the inferior temporal lobe, and the dorsolateral prefrontal cortex. Changes in GMV were observed also in dysgeusic patients, but in different brain areas (Sinding et al., 2016).

Shinozaki et al. noticed that BMS patients showed a greater number of significantly activated areas during the palm stimulation, including the left S2 cortex, left dorsolateral prefrontal cortex, left insula, left visual cortex, right posterior cingulate cortex, hippocampus, parahippocampal gyrus, cerebellum. Moreover, the lip noxious stimulation detected significantly greater activation of the left premotor cortex, left orbitofrontal cortex, mPFC, left dorsolateral prefrontal cortex, left anterior cingulate cortex, left insula, bilateral visual cortex, left caudate nucleus, and midbrain in BMS patients (Shinozaki et al., 2016).

Comparing 14 BMS patients with 14 controls, Wada et al. found that the bilateral rostral anterior cingulate cortex, the right medial orbitofrontal cortex, and the left pars orbitalis which belongs to the medial pain system, had significantly different local brain connectivity in BMS brain. Moreover, a deeper connection of the anterior cingulate cortex and mPFC with the basal ganglia, thalamus, and brain stem was observed, without differences in the somatic sensory cortex (Wada et al., 2017).

In a study comparing 27 BMS patients to 21 controls all exposed to angry facial expressions, the BMS group exhibited higher tactile ratings and greater activation in the postcentral gyrus during the presentation of tactile stimuli (Yoshino et al., 2017). In both groups the changes in brain activity elicited by angry facial images positively correlated with the changes in tactile rating scores (Yoshino et al., 2017).

Investigation of the pain modulating system to a continuous hot stimulus in 15 BMS patients and 15 controls found that in BMS this system is dysregulated and highly sensitized to pain derived from the trigeminal system. During palm stimulation, the brain regions with higher activation in BMS patients included the somatosensory areas visual cortex, cerebral limbic system, and cerebellum. During lip stimulation, the higher activation regions were the motor-related areas, cognito-affective areas, visual cortex, caudate nucleus, and midbrain (Kohashi et al., 2020).

Regarding the structural connectivity, not all the studies had convergent results. Kurokawa et al. showed that the betweenness centrality was significantly increased in the left insula, right amygdala, and right lateral orbitofrontal cortex and significantly decreased in the right inferotemporal cortex in BMS patients compare to healthy individuals (Kurokawa et al., 2021).

## Discussion

Significant differences were found between activation patterns of BMS patients and the control group in the anterior cingulate gyrus (ACC), bilateral thalamus, left lingual gyrus, bilateral precuneus, right middle frontal gyrus, right pre-central gyrus, and right inferior semilunar lobule of the cerebellum (Albuquerque et al., 2006). In BMS patients a strengthened connection of the anterior cingulate cortex and mPFC with the basal ganglia, thalamus, and brain stem was observed (Wada et al., 2017).

ACC is part of the limbic system and it is connected among other structures, with the orbitofrontal cortex, the amygdala, the parahippocampal gyrus, and with the rostral superior temporal gyrus and is associated with emotional aspects of pain sense such as evocation, choice of response, foresight, and avoidance of pain stimuli (reward value) (Rolls, 2019). Also, the cingulate cortex appeared to be specifically involved in trigeminal pain processing/modulation (Fuchs et al., 2014).

BMS patients had also increased brain GMV and lower white matter fractional anisotropy in the hippocampus and decreased GMV in mPFC (Khan et al., 2014; Tan et al., 2019). The neural mechanisms of chronic pain create an imbalance in brain regions by increasing the connectivity to salience networks and decreased connectivity to default mode networks (Kim, 2020). Decreased GMV in mPFC has been reported in a number of conditions characterized by chronic pain such as back pain, trigeminal neuralgia, temporomandibular disorder, functional dyspepsia, and also in depression and anxiety (Ong et al., 2019). Region of interest analysis suggested that the functional connectivity between the bilateral mPFC and amygdala correlated with the years of BMS (Tan et al., 2019).

fMRI data also suggests that the thalamus is hypoactive in BMS patients compared to healthy controls, a feature described in other patients with chronic pain (Khan et al., 2014), probably as a result of persistent, spontaneous chronic pain input.

These features in BMS patients emphasize that the pain modulating system is dysregulated similar to those of the patients with other neuropathic pain conditions (trigeminal neuralgia, temporomandibular disorders, herpes simplex recurrent infections, etc.) and that the brain is highly sensitized to pain information from the trigeminal system.

Nevertheless, it is significant to note that gender differences were found in the structural and functional anatomy of young adults brains: females exhibit greater GMV values, superior regional homogeneity, and stronger functional connections than males (Zhang et al., 2020). Despite this, BMS is not frequently found in young females.

In interpreting the results of the presented studies, we must take into account their limitations, such as different methods of data acquisition, processing, and analysis, low number of investigated subjects, gender disproportion of the individuals, psychophysical differences between patients and controls or insufficient psyhological evaluations, the effects of medication in BMS patients, etc.

However, although fMRI is an expensive and less accessible method, it is emerging as a useful tool in the research, diagnosis, and stratification of patients with BMS, facilitating the use of accurate therapeutic strategies.

That being said, there is a need for further studies, with a larger number of patients and in better controlled and standardized conditions.

## Author contributions

CD, IP, and REP designed the sections of the manuscript. CD, IP, and EM performed analysis and contributed to the conception and study design. REP wrote the first draft of the manuscript. CD, IP, and MD wrote sections of the manuscript. All authors contributed to the article, manuscript revision, read, and approved the submitted version.

## Funding

This work was supported by a grant of the Romanian Ministry of Research, Innovation and Digitization CCCDI - UEFISCDI, project number PN-III-P2-2.1-PED-2019-1339 within PNCDI III (Contract number 564PED/2021).

## Conflict of interest

The authors declare that the research was conducted in the absence of any commercial or financial relationships that could be construed as a potential conflict of interest.

## Publisher's note

All claims expressed in this article are solely those of the authors and do not necessarily represent those of their affiliated organizations, or those of the publisher, the editors and the reviewers. Any product that may be evaluated in this article, or claim that may be made by its manufacturer, is not guaranteed or endorsed by the publisher.

## References

[B1] Albuquerque R.J. C. de Leeuw R. Carlson C. R. Okeson J. P. Miller C. S. Andersen A. H. (2006). Cerebral activation during thermal stimulation of patients who have burning mouth disorder: an fMRI study. Pain 122, 223–234. 10.1016/j.pain.2006.01.020 16632202

[B2] Carreño-Hernández I. Cassol-Spanemberg J. Rodríguez de Rivera-Campillo E. Estrugo-Devesa A. López-López J. (2021). Is burning mouth syndrome a neuropathic pain disorder? A systematic review. J. Oral Facial Pain Headache 35, 218–229. 10.11607/ofph.2861 34609380

[B3] Eliav E. Kamran B. Schaham R. Czerninski R. Gracely R. H. Benoliel R. (2007). Evidence of chorda tympani dysfunction in patients with burning mouth syndrome. J. Am. Dental Assoc. 138, 628–633. 10.14219/jada.archive.2007.0234 17473041

[B4] Forssell H. Jääskeläinen S. Tenovuo O. Hinkka S. (2002). Sensory dysfunction in burning mouth syndrome. Pain 99, 41–47. 10.1016/S0304-3959(02)00052-0 12237182

[B5] Fuchs P. N. Peng Y. B. Boyette-Davis J. A. Uhelski M. L. (2014). The anterior cingulate cortex and pain processing. Front. Integr. Neurosci. 8:35. 10.3389/fnint.2014.00035 24829554 PMC4017137

[B6] Grushka M. Epstein J. B. Gorsky M. (2002). Burning mouth syndrome: differential diagnosis. Dermatol. Ther. 15, 287–291. 10.1046/j.1529-8019.2002.01535.x

[B7] Harel N. Ugurbil K. Uludag K. Yacoub E. (2006). Frontiers of brain mapping using MRI. J. Magn. Reson. Imaging 23, 945–957. 10.1002/jmri.20576 16649202

[B8] Imamura Y. Okada-Ogawa A. Noma N. Shinozaki T. Watanabe K. Kohashi R.. (2020). A perspective from experimental studies of burning mouth syndrome. J. Oral Sci. 62, 165–169. 10.2334/josnusd.19-0459 32161235

[B9] International Classification of Orofacial Pain 1st edition (ICOP). (2020). Cephalalgia 40, 129–221. 10.1177/0333102419893823 32103673

[B10] International Headache Society (2018). Headache Classification Committee of the International Headache Society (IHS) The International Classification of Headache Disorders, 3rd edition. Cephalalgia 38, 1–211. 10.1177/0333102417738202 29368949

[B11] Jasanoff A. (2007). Bloodless fMRI. Trends Neurosci. 30, 603–610. 10.1016/j.tins.2007.08.002 17935797

[B12] Khan S. A. Keaser M. L. Meiller T. F. Seminowicz D. A. (2014). Altered structure and function in the hippocampus and medial prefrontal cortex in patients with burning mouth syndrome. Pain 155, 1472–1480. 10.1016/j.pain.2014.04.022 24769366

[B13] Kim J. (2020). Somatotopically specific primary somatosensory connectivity to salience and default mode networks encodes clinical pain. J. Acupunct. Meridian Stud. 13, 74. 10.1016/j.jams.2020.03.023 PMC6586503 30839429

[B14] Kohashi R. Shinozaki T. Sekine N. Watanabe K. Takanezawa D. Nishihara C.. (2020). Time-dependent responses in brain activity to ongoing hot stimulation in burning mouth syndrome. J. Oral Sci. 62, 170–174. 10.2334/josnusd.18-0431 32224570

[B15] Kurokawa R. Kamiya K. Inui S. Kato S. Suzuki F. Amemiya S.. (2021). Structural connectivity changes in the cerebral pain matrix in burning mouth syndrome: a multi-shell, multi-tissue-constrained spherical deconvolution model analysis. Neuroradiology 63, 2005–2012. 10.1007/s00234-021-02732-9 34142212

[B16] Logothetis N. K. (2008). What we can do and what we cannot do with fMRI. Nature 453, 869–878 10.1038/nature06976 18548064

[B17] Nagamine T. (2022). Two-hit theory by estrogen in burning mouth syndrome. *J. Dental Sci*. 10.1016/j.jds.2022.06.009. Available online at: https://www.sciencedirect.com/science/article/pii/S1991790222001416?via%3Dihub PMC9588802 36299303

[B18] Ong W. Y. Stohler C. S. Herr D. R. (2019). Role of the prefrontal cortex in pain processing. Mol. Neurobiol. 56, 1137–1166. 10.1007/s12035-018-1130-9 29876878 PMC6400876

[B19] Orliaguet M. Misery L. (2021). Neuropathic and psychogenic components of burning mouth syndrome: a systematic review. Biomolecules 11, 1237. 10.3390/biom11081237 34439903 PMC8393188

[B20] Rolls E. T. (2019). The cingulate cortex and limbic systems for emotion, action, and memory. Brain Struct. Funct. 224, 3001–3018. 10.1007/s00429-019-01945-2 31451898 PMC6875144

[B21] Scala A. Checchi L. Montevecchi M. Marini I. Giamberardino M. A. (2003). Update on burning mouth syndrome: overview and patient management. Crit. Rev. Oral Biol. Med. 14, 275–291. 10.1177/154411130301400405 12907696

[B22] Seol S.-H. Chung G. (2022). Estrogen-dependent regulation of transient receptor potential vanilloid 1 (TRPV1) and P2X purinoceptor 3 (P2X3): implication in burning mouth syndrome. J. Dental Sci. 17, 8–13. 10.1016/j.jds.2021.06.007 35028015 PMC8739235

[B23] Shinozaki T. Imamura Y. Kohashi R. Dezawa K. Nakaya Y. Sato Y.. (2016). Spatial and temporal brain responses to noxious heat thermal stimuli in burning mouth syndrome. J. Dent. Res. 95, 1138–1146. 10.1177/0022034516653580 27302878

[B24] Sinding C. Gransjøen A. M. Schlumberger G. Grushka M. Frasnelli J. Singh P. B. (2016). Grey matter changes of the pain matrix in patients with burning mouth syndrome. Eur. J. Neurosci. 43, 997–1005. 10.1111/ejn.13156 26741696

[B25] Tan Y. Wu X. Chen J. Kong L. Qian Z. (2019). Structural and functional connectivity between the amygdala and orbital frontal cortex in burning mouth syndrome: an fMRI study. Front. Psychol. 10, 1700. 10.3389/fpsyg.2019.01700 PMC6669911 31404248

[B26] Van I. (2021). Burning Mouth Disease: A Guide to Diagnosis and Management. Cham, Switzerland: Springer Nature.

[B27] Wada A. Shizukuishi T. Kikuta J. Yamada H. Watanabe Y. Imamura Y.. (2017). Altered structural connectivity of pain-related brain network in burning mouth syndrome—investigation by graph analysis of probabilistic tractography. Neuroradiology 59, 525–532. 10.1007/s00234-017-1830-2 28361345

[B28] Wu S. Zhang W. Yan J. Noma N. Young A. Yan Z. (2021). Worldwide prevalence estimates of burning mouth syndrome: a systematic review and meta-analysis. Oral Dis. 28, 1431–1440. 10.1111/odi.13868 33818878

[B29] Yoshino A. Okamoto Y. Doi M. Okada G. Takamura M. Ichikawa N.. (2017). Functional alterations of postcentral gyrus modulated by angry facial expressions during intraoral tactile stimuli in patients with burning mouth syndrome: a functional magnetic resonance imaging study. Front. Psychiatry. 8:224. 10.3389/fpsyt.2017.00224 29163243 PMC5681843

[B30] Zhang X. Liang M. Qin W. Wan B. Yu C. Ming D. (2020). Gender differences are encoded differently in the structure and function of the human brain revealed by multimodal MRI. Front. Hum. Neurosci. 14:244. 10.3389/fnhum.2020.00244 32792927 PMC7385398

