# Supplementary material for: Insights on brain functions in burning mouth syndrome
Source: Front Syst Neurosci. 2022 Sep 2;16:975126. doi: 10.3389/fnsys.2022.975126 (PMC9478342; doi:10.3389/fnsys.2022.975126)
Supplement: Supplementary file 1 [file Table_1.DOCX]

**Table 1.** fMRI studies results on burning mouth syndrome

| **References** | **Sample size** | **Study design** | **Outcome assessment** | **Conclusions** |
| --- | --- | --- | --- | --- |
| Albuquerque et al., 2006 | Group 1: BMS, n = 8 females, mean age 49.1 ± 10.1  Group 2: n = 8 healthy females, mean age 50.3 ± 12.3 | Thermal stimulation of the trigeminal nerve mapped using fMRI | BMS patients showed greater fractional signal changes in the right anterior cingulate cortex (BA 32/24) and bilateral precuneus. | Patients with BMS show brain activation patterns resembling those of patients suffering from other types of chronic neuropathic pain disorders. |
| Khan et al., 2014 | Group 1: BMS, n = 9 females  Group 2: n = 9 healthy females | Brain gray matter volume (GMV), white matter fractional anisotropy (FA), and functional connectivity. | BMS patients showed increased GMV and lower FA in the hippocampus (Hc), and decreased GMV in the medial prefrontal cortex (mPFC) | Evidence of dysfunctional structure and function of mPFC and Hc, and their implication in regulating mood and depressive symptoms in BMS |
| Sinding et al., 2016 | Group 1: idiopathic BMS, n = 12, 7 women, 35–72 years, mean age 59.4 ± 12.1 years  Group 2: Dysgeusic patients, n = 17, 11 women, 42–73 years, 58.4 ± 8.1 years  Group 3: healthy subjects n = 13, 10 women, 50–73 years, 59 ±3.4 years | Gray matter concentration via MRI. | In BMS patients, a deficiency in the control of pain, could in part explain cardinal BMS symptoms and suggests a central pain condition or main component. | A deficiency in the control of pain could in part be a cause of BMS and suggests a central pain condition. |
| Shinozaki et al., 2016 | Group 1: BMS, n = 16 females, under 65 years  Group 2: n = 15 healthy females, mean age 50.3 ± 12.3, under 65 years | Brain response via fMRI data to noxious heat stimuli on the right palm or right lower lip. | Greater activation from noxious stimulation on the lip in BMS patients validated the hypothesis that the BMS symptomatology is based on mechanisms that affect the central and peripheral nervous systems. | BMS patients show specific brain responses due to impaired function of the central and peripheral nervous  systems. |
| Wada et al., 2017 | Group 1: BMS, n = 14 females, aged 42–63 years (mean age 50.9 years)  Group 2: n = 14 females, 42–65 years (mean age 50.2 years) | Brain network of BMS brain by using probabilistic tracto-graphy and graph analysis. | In primary BMS patients, structural brain network analysis via fMRI revealed significant alteration of the medial system of the pain-related brain network. | Structural brain network analysis reveals the alteration of the medial system of the pain-related brain network in chronic pain syndrome. |
| Yoshino et al., 2017 | Group 1: BMS, n=27 patients (21 women, mean age = 44.8 ± 12.0 years)  Group 2: 21 gender- and age-matched control subjects (18 women, mean age = 46.3 ± 10.7 years). | Activation of brain regions in response to intraoral tactile stimuli when modulated by angry facial expressions. | BMS patients showed more somatosensory changes in activation induced by anger-context tactile stimuli than in controls. | The interaction between brain activity and emotional context associated with tactile stimuli may play an important role in the pathophysiology of BMS. |
| Tan et al., 2019 | Group 1: BMS, n=26 patients (21 women, mean age = 52.12 ± 8.81 years)  Group 2: 27 gender- and age-matched control subjects (25 women, mean age = 51.11 ± 5.42 years) | Structural and functional connectivity between the amygdala and orbital frontal cortex via fMRI | The severity of BMS was associated with the degree of increased functional connectivity between bilateral ventromedial prefrontal cortex (VMPFC) and the bilateral amygdala and lowerGMV in VMPFC. | The increased functional connectivity between the VMPFC and amygdala observed in BMS patients is associated with the severity of the disease. |
| Kohashi et al., 2020 | Group 1: BMS, 15 right-handed female patients (52.6 ± 6.3 years)  Group 2: 15 age- and gender matched, right-handed female controls (49.0 ± 8.4 years). | Temporal brain responses to an ongoing hot stimulus to investigate the pain modulating system via fMRI. | The brain in patients with BMS was highly sensitized to pain signals originating from the trigeminal system. Small nerve fiber atrophy was observed in the oral mucosa of the BMS patients. | Dysregulation of pain modulating system in BMS patients. BMS brain is highly sensitized to pain information which originates from the trigeminal system.. |
| Kurokawa et al., 2021 | Group 1: BMS, n = 14 subjects  Group 2: n = 11 healthy subjects | Structural connectivity changes in the cerebral pain matrix in BMS | BMS patients had alterations in the regions that comprise the pain matrix and medial pain ascending pathway. These results highlighted the emotional-affective profile of BMS, which is a chronic pain syndrome. | BMS patients show alterations in the regions comprising the pain matrix and medial pain ascending pathway. |
